# High Ambient Temperature Represses Anthocyanin Biosynthesis through Degradation of HY5

**DOI:** 10.3389/fpls.2017.01787

**Published:** 2017-10-20

**Authors:** Sara Kim, Geonhee Hwang, Seulgi Lee, Jia-Ying Zhu, Inyup Paik, Thom Thi Nguyen, Jungmook Kim, Eunkyoo Oh

**Affiliations:** ^1^Department of Bioenergy Science and Technology, Chonnam National University, Gwangju, South Korea; ^2^Department of Plant Biology, Carnegie Institution for Science, Stanford, CA, United States; ^3^Department of Molecular Biosciences, The Institute for Cellular and Molecular Biology, University of Texas, Austin, TX, United States

**Keywords:** *Arabidopsis*, high temperature stress, anthocyanin, HY5, flavonoid, gene expression

## Abstract

Anthocyanins are flavonoid compounds that protect plant tissues from many environmental stresses including high light irradiance, freezing temperatures, and pathogen infection. Regulation of anthocyanin biosynthesis is intimately associated with environmental changes to enhance plant survival under stressful environmental conditions. Various factors, such as UV, visible light, cold, osmotic stress, and pathogen infection, can induce anthocyanin biosynthesis. In contrast, high temperatures are known to reduce anthocyanin accumulation in many plant species, even drastically in the skin of fruits such as grape berries and apples. However, the mechanisms by which high temperatures regulate anthocyanin biosynthesis in *Arabidopsis thaliana* remain largely unknown. Here, we show that high ambient temperatures repress anthocyanin biosynthesis through the E3 ubiquitin ligase CONSTITUTIVE PHOTOMORPHOGENIC1 (COP1) and the positive regulator of anthocyanin biosynthesis ELONGATED HYPOCOTYL5 (HY5). We show that an increase in ambient temperature decreases expression of genes required in both the early and late steps of the anthocyanin biosynthesis pathway in *Arabidopsis* seedlings. As a result, seedlings grown at a high temperature (28°C) accumulate less anthocyanin pigment than those grown at a low temperature (17°C). We further show that high temperature induces the degradation of the HY5 protein in a COP1 activity-dependent manner. In agreement with this finding, anthocyanin biosynthesis and accumulation do not respond to ambient temperature changes in *cop1* and *hy5* mutant plants. The degradation of HY5 derepresses the expression of *MYBL2*, which partially mediates the high temperature repression of anthocyanin biosynthesis. Overall, our study demonstrates that high ambient temperatures repress anthocyanin biosynthesis through a COP1-HY5 signaling module.

## Introduction

**FIGURE 1:**
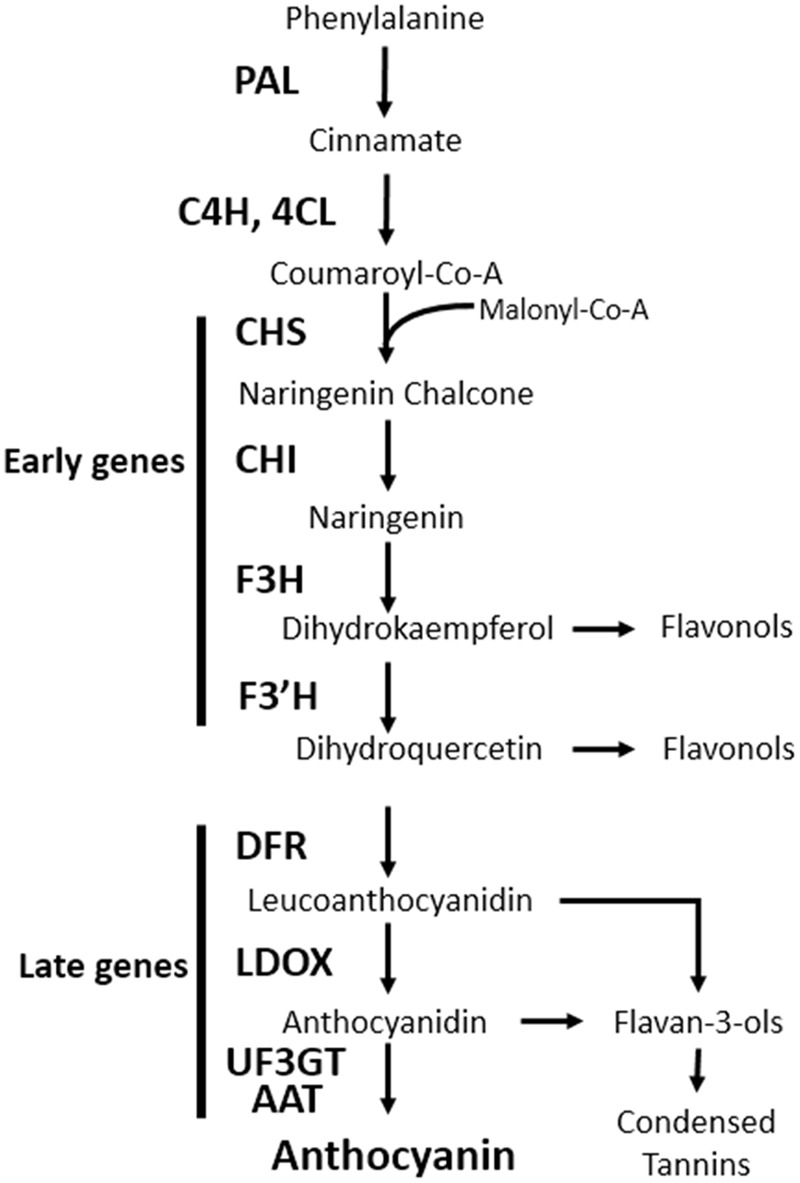
Schematic diagram of the anthocyanin biosynthetic pathway in *Arabidopsis thaliana*. PAL, phenylalanine ammonia lyase; C4H, cinnamate 4-hydroxylase; 4CL, 4-coumaroyl: CoA-ligase; CHS, chalcone synthase; CHI, chalcone isomerase; F3H, flavanone 3-hydroxylase; F3′H, flavonoid 3′-hydroxylase; DFR, dihydroflavonol reductase; LDOX, leucoanthocyanidin dioxygenase; UF3GT, UDP-glucose flavonoid 3-*O*-glucosyltransferase; AAT, anthocyanin acyltransferase.

Anthocyanins are water-soluble pigments found throughout the plant kingdom and are derived from the flavonoid branch of the phenylpropanoid pathway. Anthocyanins play key roles in diverse physiological processes, such as protecting plant tissues from damage after exposure to UV, freezing temperature, and pathogens; assist in the attraction of pollinators and seed dispersers, scavenge free radicals produced under stress conditions, and modulate auxin transport (Falcone Ferreyra et al., 2012). The anthocyanin biosynthesis pathway has been studied in numerous plant species and most of the genes involved in this process have been identified. In the model plant *Arabidopsis thaliana*, the genes encoding anthocyanin biosynthesis enzymes are grouped into two classes: early biosynthetic genes [*chalcone synthase* (*CHS*), *chalcone isomerase* (*CHI*), *flavanone 3-hydroxylase* (*F3H*), and *flavonoid 3′-hydroxylase* (*F3′H*)], all of which are common to other flavonoids; and late biosynthetic genes [*dihydroflavonol 4-reductase* (*DFR*), *leucoanthocyanidin oxygenase* (*LDOX*), *UDP-glucose flavonoid 3-O-glucosyltransferase* (*UF3GT*), and *anthocyanin acyltransferase* (*AAT*)] that are specific to the anthocyanin pathway (**Figure 1**) (Tanaka et al., 2008).

Anthocyanin pigment accumulation is mainly regulated via the transcriptional regulation of anthocyanin biosynthetic genes (Tanaka et al., 2008). Several transcription factors that regulate the expression of anthocyanin biosynthetic genes have been identified. The R2R3-type MYB transcription factors PAP1/MYB75, PAP2/MYB90, MYB113, and MYB114 positively regulate anthocyanin biosynthesis in vegetative tissues (Borevitz et al., 2000; Teng et al., 2005; Gonzalez et al., 2008). Three members of the bHLH transcription factor subgroup IIIf, GLABRA3 (GL3), ENHANCER OF GLABRA3 (EGL3), and TRANSPARENT TESTA8 (TT8), are positive regulators of anthocyanin biosynthesis (Zhang et al., 2003). The bHLH and MYB transcription factors form a ternary complex with the WD40 protein TTG1 to activate anthocyanin biosynthesis, particularly the late biosynthetic steps (Zhang et al., 2003; Gonzalez et al., 2008). In contrast, the small MYB protein MYBL2 negatively regulates anthocyanin biosynthesis by repressing the expression of the late biosynthetic genes *DFR* and *LDOX* (Dubos et al., 2008; Matsui et al., 2008).

Since anthocyanin biosynthesis is a metabolically expensive process, it is closely regulated with respect to surrounding environmental conditions. Thus, the regulation of anthocyanin biosynthetic genes is associated with many environmentally responsive signaling pathways. For example, UV, visible, and far-red light irradiation can induce anthocyanin biosynthesis. Photoreceptors including UV-B RESISTANCE8 (UVR8), phytochromes (PHYs), and cryptochromes (CRYs) mediate light induction of anthocyanin biosynthesis (Sponga et al., 1986; Mancinelli et al., 1991; Heijde et al., 2013). The ring-finger E3 ubiquitin ligase CONSTITUTIVE PHOTOMORPHOGENIC1 (COP1) regulates anthocyanin biosynthesis downstream of the photoreceptors. COP1 has been shown to induce the degradation of PAP1 and PAP2 to repress anthocyanin biosynthesis in the absence of light (Maier et al., 2013). COP1 also interacts with a bZIP transcription factor, LONG HYPOCOTYL5 (HY5), that directly binds to the promoters of both early and late anthocyanin biosynthetic genes and activates their expression (Chattopadhyay et al., 1998; Shin et al., 2007). In addition to light, anthocyanin accumulation is also induced by cold stress, a response partly mediated by HY5 (Catala et al., 2011). Osmotic stress, nitrogen deficiency, and pathogen infection have also been shown to induce anthocyanin biosynthesis (Lea et al., 2007; Tanaka et al., 2008). In addition, it has been shown that anthocyanin accumulation is affected by ambient temperature in various plant species. For example, anthocyanin accumulation in the skins of ripening fruit (grapevine and apple) is reduced in response to high temperatures (Mori et al., 2007; Lin-Wang et al., 2011; Movahed et al., 2016; Pastore et al., 2017). However, the effects of ambient temperature changes on anthocyanin biosynthesis in *Arabidopsis*, are poorly understood.

Temperature-related changes in plant development and growth are critical for survival in a variable environment. For example, young seedlings respond to increases in ambient temperature by elongation of hypocotyls and petioles, hyponastic leaf growth, and leaf thinning; these responses are collectively called thermomorphogenesis (Quint et al., 2016). The morphological changes are considered to increase plant survival under heat stress, in part by enhancing leaf transpiration efficiency (Crawford et al., 2012; Zhu et al., 2016). The bHLH transcription factor PHYTOCHROME INTERACTING FACTOR4 (PIF4) is a key regulator of thermomorphogenesis (Koini et al., 2009; Proveniers and van Zanten, 2013; Choi and Oh, 2016). HY5 has also been reported to negatively regulate thermomorphogenic growth (Delker et al., 2014). Although thermomorphogenesis, especially hypocotyl elongation, has been extensively studied, it is still not clear how high ambient temperatures impinge on other physiological processes including anthocyanin biosynthesis.

In this study, we show that a high temperature represses anthocyanin biosynthesis through HY5 in *Arabidopsis*. Elevation of ambient temperature decreases the expression of several anthocyanin biosynthetic genes and results in a lower level of anthocyanin accumulation in *Arabidopsis* seedlings. HY5 and COP1 both appear to be required for the thermo-sensitivity of anthocyanin accumulation and expression of anthocyanin biosynthetic genes. We also show that the level of the HY5 protein, but not the level of *HY5* mRNA, is reduced by high temperature in a COP1 activity-dependent manner. Taken together, our study demonstrates that the control of anthocyanin biosynthesis in response to high ambient temperature changes is largely exerted through degradation of HY5.

## Results

### High Temperature Represses the Accumulation of Anthocyanin Pigments

**FIGURE 2:**
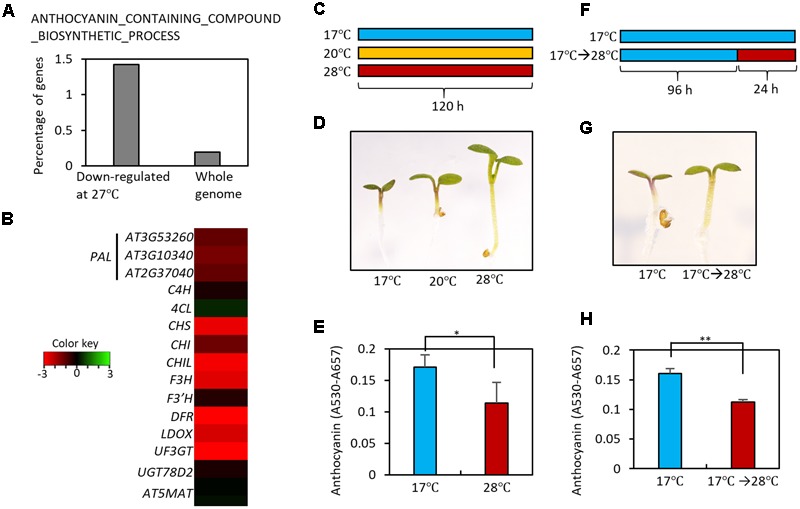
High temperature represses the accumulation of anthocyanin pigments. **(A)** Gene Ontology (GO) analyses showed that genes in the ANTHOCYANIN_CONTAINING_COMPOUND_BIOSYNTHETIC_PROCESS are highly enriched among high temperature (27°C)-repressed genes identified in a previous RNA-seq experiment (Jung et al., 2016). Other molecular processes enriched in the high temperature-repressed genes are shown in Supplementary Table 1. **(B)** Heat map of the anthocyanin biosynthetic genes at Zeitgeber Time 0 (ZT0) in previous RNA-seq experiment (Jung et al., 2016). Red color indicates genes that were down-regulated by high temperatures and green color indicates genes that were up-regulated by high temperatures. **(C)** Growth conditions in experiments **(D,E)**. Seedlings were grown on MS medium containing 3% sucrose at different temperatures (17, 20, or 28°C) for 5 days under continuous white light, and then harvested for anthocyanin extraction. **(D,E)** A lower level of anthocyanin accumulation was found in seedlings grown at the higher temperatures. Representative seedlings are shown in **(D)** and quantification of anthocyanin content is shown in **(E)**. Error bars in **(E)** indicate s.d. (*n* = 3). ^∗^*P* < 0.05 (Student’s *t*-test). **(F)** Growth conditions in experiments **(G,H)**. Seedlings were either grown at 17°C for 4 days and then at 28°C for 24 h before harvesting, or were kept at 17°C as a control. **(G,H)** Anthocyanin accumulation was reduced by the high temperature treatment. Representative seedlings are shown in **(G)** and quantification of anthocyanin content is shown in **(H)**. Error bars in **(H)** indicate s.d. (*n* = 3). ^∗∗^*P* < 0.01 (Student’s *t*-test).

In order to identify developmental responses to an elevation in ambient temperature, we performed gene ontology (GO) analyses using high temperature-regulated genes identified in a previous RNA-sequencing experiment (Jung et al., 2016). These high temperature-regulated genes were identified by comparing the transcriptomes of wild type (Ler) seedlings grown at 22°C with those of wild type seedlings grown at 27°C at Zeitgeber Time (ZT) 0, 1, 4, 8, 12, 16, 20, and 22 h. The GO analyses revealed that genes for the biosynthesis of anthocyanin-containing compounds were highly enriched in the high temperature-repressed genes at ZT0 (**Figure 2A** and Supplementary Table 1). Most anthocyanin biosynthetic genes, including both early and late genes, were transcriptionally repressed in seedlings grown at 27°C compared to those grown at 20°C (**Figure 2B**). These results suggest that high ambient temperatures may directly repress anthocyanin pigment accumulation. To test this hypothesis, we grew *Arabidopsis* seedlings on MS medium containing 3% sucrose at different temperatures (17, 20, and 28°C) under continuous light (**Figure 2C**). As reported previously, the increase in ambient temperature to 28°C dramatically induced hypocotyl elongation (**Figure 2D**) (Koini et al., 2009). In addition to this known morphological change, we also observed that anthocyanin pigment accumulation was lower in these seedlings than in seedlings grown at 17 or 20°C (**Figures 2D,E**). To determine whether anthocyanin pigment accumulation was affected by short and transient ambient temperature changes, seedlings were grown for 4 days at 17°C and then transferred to 28°C for 24 h (**Figure 2F**). Anthocyanin pigment levels in seedlings exposed to 28°C for 24 h were significantly lower than in seedlings kept at 17°C (**Figures 2G,H**). These results indicate that high ambient temperature represses anthocyanin pigment accumulation in young seedlings.

### High Temperature Transcriptionally Represses Anthocyanin Biosynthetic Genes

**FIGURE 3:**
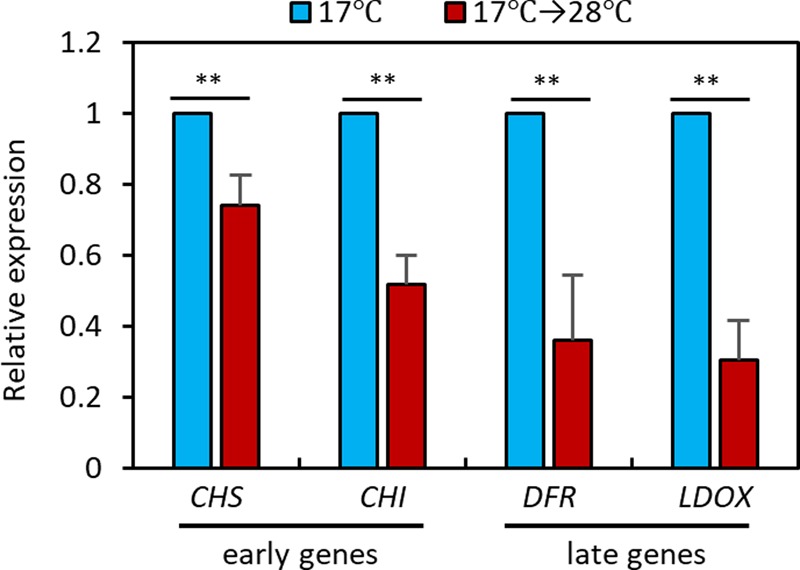
High temperature represses expression of anthocyanin biosynthetic genes. The qRT-PCR analyses showed that expression of anthocyanin biosynthetic genes was decreased at 28°C. Wild type seedlings were grown at 17°C under continuous white light for 4 days and then either transferred to 28°C for 24 h (17°C → 28°C) or kept at 17°C before harvesting for total RNA extraction. Gene expression levels were normalized to *PP2A* (AT1G13320) and are presented as values relative to those of seedlings kept at 17°C. Error bars indicate s.d. (*n* = 3). ^∗∗^*P* < 0.01 (Student’s *t*-test).

To elucidate the molecular mechanisms underlying the high temperature repression of anthocyanin accumulation, we performed a qRT-PCR analysis to examine the expression levels of anthocyanin biosynthesis genes in seedlings grown at 28°C for 24 h or kept at 17°C. The qRT-PCR analysis revealed that expression of the early anthocyanin biosynthetic genes *CHS* and *CHI* was lower by about 20 to 40% in seedlings exposed to 28°C than in those kept at 17°C (**Figure 3**). The late anthocyanin biosynthesis genes *DFR* and *LDOX* also showed a twofold reduction in expression after the high temperature treatment (**Figure 3**). The expression patterns of the anthocyanin biosynthetic genes were consistent with the observation of lower levels of anthocyanin pigments in seedlings grown at 28°C than in those grown at 17°C (**Figure 2**), indicating that the decreased anthocyanin accumulation induced by the elevated ambient temperature was caused by transcriptional reduction of both early and late anthocyanin biosynthetic genes.

### HY5 Mediates the High Temperature Regulation of Anthocyanin Biosynthesis

**FIGURE 4:**
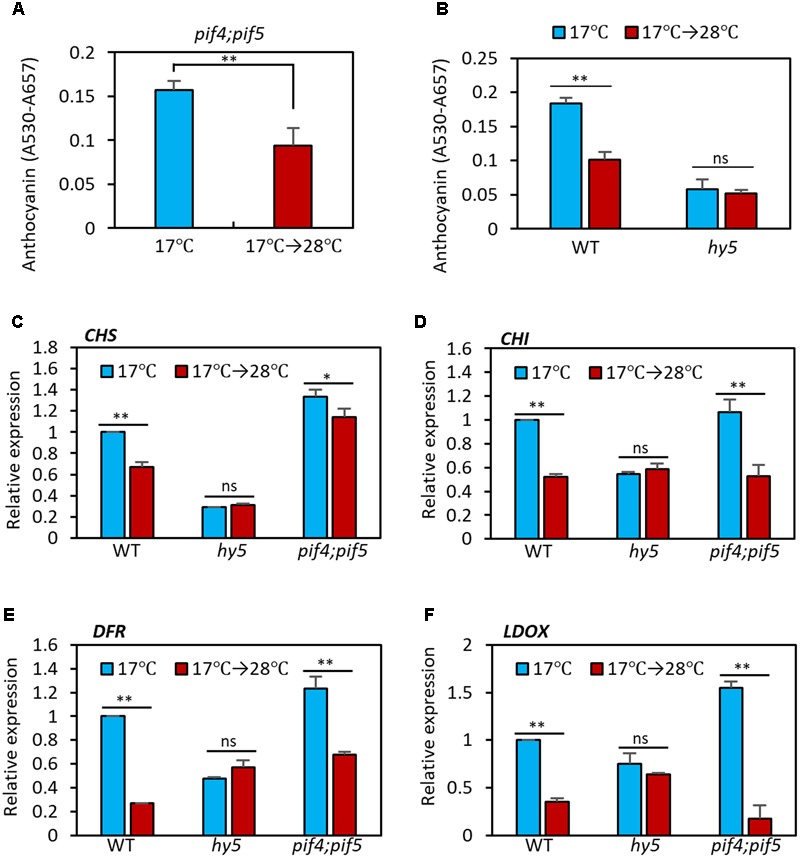
Anthocyanin biosynthesis is insensitive to high temperature in *hy5* mutant. **(A)** Anthocyanin accumulation was repressed by high temperature treatment in *pif4;pif5* double mutant seedlings. The *pif4;pif5* seedlings were grown at 17°C under continuous white light for 4 days and then either transferred to 28°C for 24 h (17°C → 28°C) or kept at 17°C before harvesting for anthocyanin extraction. **(B)** Anthocyanin accumulation in the *hy5* mutant was not significantly affected by an increase in ambient temperature. Seedlings of wild type and *hy5* mutant were grown under the same conditions as **(A)**. Error bars in **(A,B)** indicate s.d. (*n* = 3). ^∗∗^*P* < 0.01 (Student’s *t*-test); ns, not significant (*P* ≥ 0.05). **(C–F)** The qRT-PCR analyses showed that expression of both early and late anthocyanin biosynthetic genes were insensitive to high temperature in the *hy5* mutant. Seedlings were grown at 17°C for 4 days and then transferred to 28°C for 24 h (17°C → 28°C) or kept at 17°C before harvesting for total RNA extraction. Gene expression levels were normalized to *PP2A* (AT1G13320) and are presented as values relative to those of wild type seedlings kept at 17°C. Error bars indicate s.d. (*n* = 3). ^∗∗^*P* < 0.01 (Student’s *t*-test) and ns, not significant (Student’s *t*-test *P* ≥ 0.05).

PIF4 is a key transcription factor in high temperature acclimation processes including thermomorphogenesis and acceleration of flowering (Choi and Oh, 2016; Quint et al., 2016). Moreover, it was recently reported that PIF4, and its closest homolog PIF5, negatively regulate anthocyanin biosynthesis under red light (Liu et al., 2015). Thus, it was likely that PIF4 mediated the high temperature regulation of anthocyanin biosynthesis. To test whether this was the case, we measured anthocyanin pigment levels in *pif4;pif5* double mutant seedlings grown at 17°C or at 17°C for 4 days followed by 28°C for 24 h. As shown in **Figure 4A**, anthocyanin levels in *pif4;pif5* seedlings were significantly reduced by the high temperature treatment in a similar manner to that in the wild type, suggesting that PIF4 and PIF5 are not major factors that mediate the high temperature repression of anthocyanin biosynthesis.

The bZIP transcription factor HY5 is a positive regulator of anthocyanin biosynthesis and directly regulates expression of several anthocyanin biosynthetic genes, including both early and late genes (Shin et al., 2007, 2013). HY5 also regulates the expression of anthocyanin-promoting transcription factors such as *PAP1* (Shin et al., 2013). Moreover, HY5 has previously been reported to positively regulate cold-induced gene expression and cold acclimation and negatively regulate high temperature-induced hypocotyl elongation (Catala et al., 2011; Delker et al., 2014). We therefore examined whether HY5 mediates the high temperature repression of anthocyanin biosynthesis. As previously reported (Shin et al., 2007), anthocyanin pigment levels were lower in *hy5* mutant seedlings at 17°C than in the wild type (**Figure 4B**). However, in contrast to the wild type, anthocyanin levels were not significantly affected by high temperature exposure in the *hy5* mutant (**Figure 4B**), supporting the idea that HY5 plays a dominant role in ambient temperature regulation of anthocyanin biosynthesis.

Next, we examined the transcriptional responses of anthocyanin biosynthetic genes to an increase in ambient temperature in wild type, *hy5*, and *pif4;pif5* seedlings. In the wild type and *pif4;pif5* seedlings, expression of *CHS* and *CHI* (early genes) was reduced about 20–40% by the high temperature treatment and expression of *DFR* and *LDOX* (late genes) was reduced by about 60–80% (**Figures 4C–F**). The expression of *UF3GT* was also dramatically decreased by the high temperature in wild type (Supplementary Figure 1). In contrast, in the *hy5* mutant, none of these genes showed any significant difference in level of expression after the high temperature treatment (**Figures 4C–F** and Supplementary Figure 1). The expression patterns of these four anthocyanin biosynthetic genes were consistent with the significant reduction in anthocyanin pigment accumulation after high temperature in the wild type and *pif4;pif5* seedlings, and also with the thermo-insensitivity of the *hy5* mutant (**Figure 4B**). Thus, HY5 appears to mediate the transcriptional regulation of anthocyanin biosynthesis genes in response to an ambient temperature change.

### High Temperature Induces HY5 Protein Degradation

**FIGURE 5:**
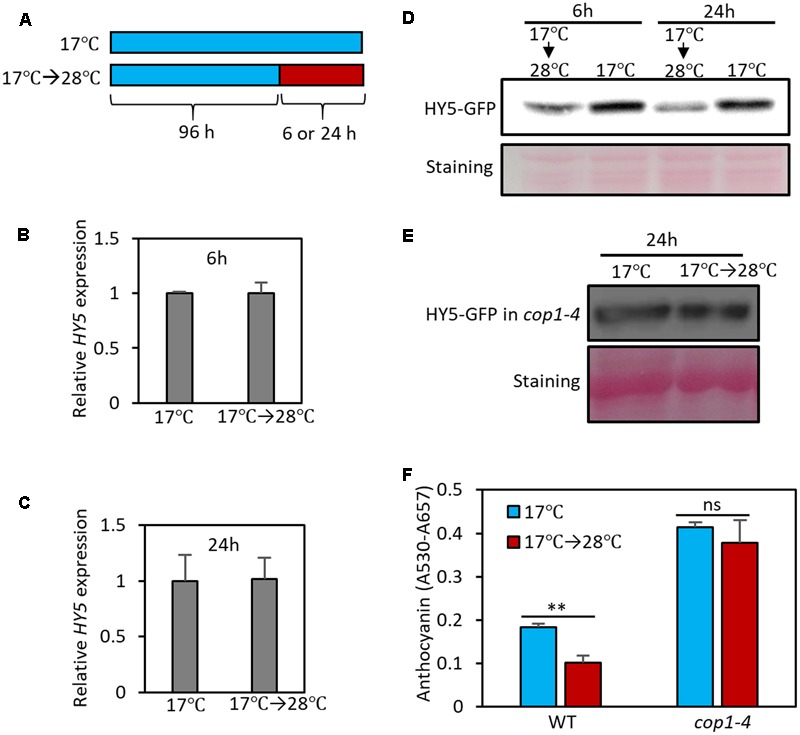
High temperature induces the degradation of HY5 protein. **(A)** Growth conditions in experiments **(B–F)**. Seedlings were grown on MS medium containing 3% sucrose at 17°C for 4 days and then transferred to 28°C for 6 or 24 h (17°C → 28°C) or kept at 17°C. The seedlings were then harvested for extraction of total RNA, protein, and anthocyanin. **(B,C)** The expression of *HY5* was not significantly affected by the 6- or 24-h high temperature treatment. Gene expression levels were normalized to *PP2A* (AT1G13320) and are presented as values relative to that of seedlings kept at 17°C. Error bars indicate s.d. (*n* = 3). **(D,E)** Western blotting with anti-GFP antibody shows that the stability of HY5 protein is reduced at high temperature in wild type, but not in *cop1-4* mutant. Total protein was extracted from *35S::HY5-GFP* **(D)** or *35S::HY5-GFP;cop1-4* **(E)** mutant plants. Equal loading of samples is shown by Ponceau S staining. **(F)** Anthocyanin pigment accumulation was not affected by high temperature (24 h) in *cop1-4* mutant seedlings. Error bars indicate s.d. (*n* = 3). ^∗∗^*P* < 0.01 (Student’s *t*-test) and ns, not significant (Student’s *t*-test *P* ≥ 0.05).

Given that HY5 is a positive regulator of anthocyanin biosynthesis, the observation that high temperature represses anthocyanin biosynthesis suggests that HY5 activity is decreased as temperature increases. To identify the molecular mechanism(s) involved in the high temperature reduction of HY5 activity, we first determined whether high temperature controls HY5 at the transcriptional level. Although a previous study reported that the level of *HY5* mRNA was decreased at high temperature (Delker et al., 2014), we did not find any significant change in *HY5* expression in response to 6 or 24 h at 28°C under our growth conditions (**Figures 5A–C**), implicating that high temperature represses HY5 protein activity post-transcriptionally. Consistent with our observation, *HY5* expression was not discernibly affected by high temperatures in a recent study (Park et al., 2017). Next, we examined the effect of high temperature on HY5 protein stability. To investigate protein stability, we measured HY5-GFP levels (driven by a *35S* promoter) by western blot analysis. This analysis showed that the HY5 protein level was slightly decreased after 6 h of high-temperature treatment and was further decreased after 24 h (**Figure 5D**). Since *HY5* mRNA levels were not significantly affected by high temperature (**Figures 5B,C**), these results suggest that high ambient temperature represses the anthocyanin biosynthesis mainly through destabilization of HY5 protein.

HY5 protein stability is known to be regulated by light through E3 ligase COP1-mediated degradation (Osterlund et al., 2000). In the dark, nuclear-localized COP1 directly interacts with HY5 and induces ubiquitination and degradation of the protein; as a result, HY5 protein levels are low in the dark. In contrast, in the light, COP1 is translocated to the cytosol by light-activated signals, resulting in HY5 protein accumulation in the nucleus (Osterlund et al., 2000). To examine whether COP1 mediates high temperature-induced HY5 degradation, we determined HY5-GFP levels in the *cop1* loss-of-function mutant (*cop1-4*) background. In contrast to wild type plants, the HY5 protein level was not significantly decreased by exposure to high temperature in the *cop1-4* mutant (**Figure 5E**), indicating that COP1 activity is required for high temperature-induced HY5 degradation. Consistent with the patterns of HY5 protein, the anthocyanin levels of *cop1-4* mutant seedlings were higher than those of wild type and were thermo-insensitive (**Figure 5F**). These results suggest that COP1 is involved in the high temperature repression of anthocyanin by destabilizing the HY5 protein.

### *MYBL2* Is Transcriptionally Induced at High Temperatures

**FIGURE 6:**
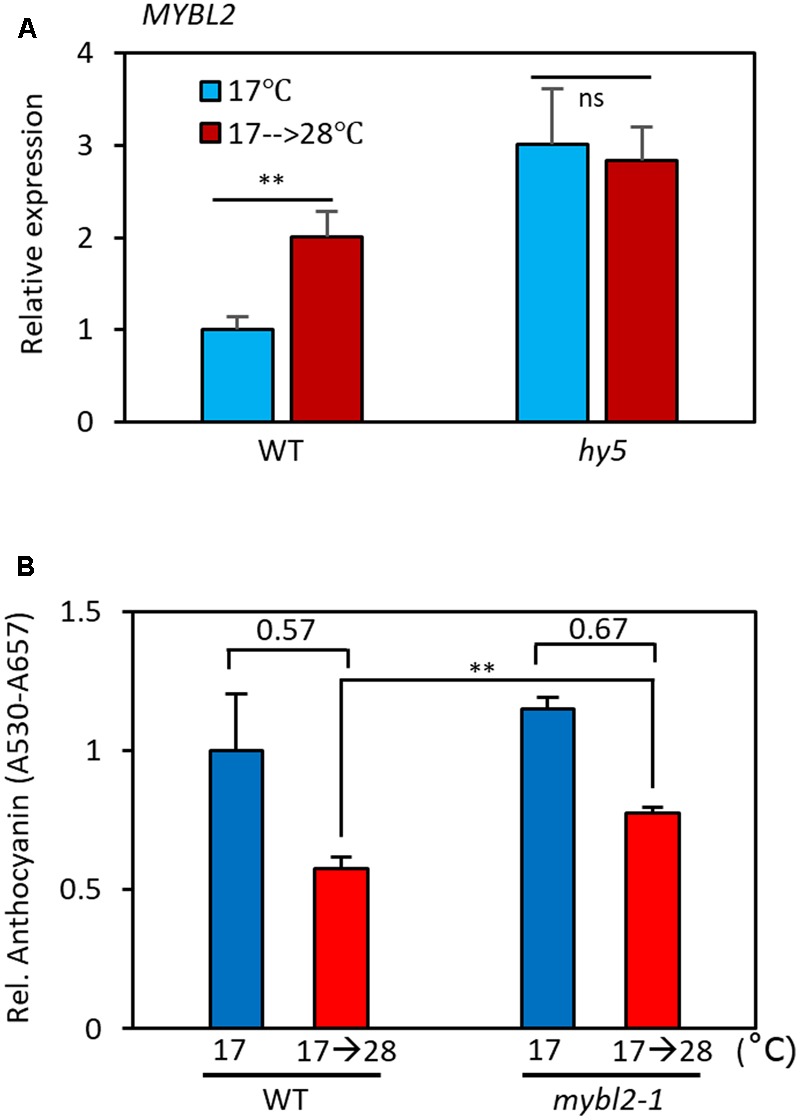
MYBL2 partially mediates the high temperature repression of anthocyanin biosynthesis. **(A)** Expression of *MYBL2* in wild type and *hy5* mutant seedlings grown at 17°C for 4 days and then transferred to 28°C for 24 h (17°C → 28°C) or kept at 17°C. *MYBL2* expression levels were normalized to *PP2A* (AT1G13320) and are presented as values relative to that of wild type seedlings kept at 17°C. Error bars indicate s.d. (*n* = 3) and ns, not significant (Student’s *t*-test *P* ≥ 0.05). **(B)** Anthocyanin levels of wild type and *mybl2* mutant seedlings grown at two different temperatures. The wild type and *mybl2-1* mutant seedlings were grown at 17°C under continuous white light for 4 days and then either transferred to 28°C for 24 h (17°C → 28°C) or kept at 17°C before harvesting for anthocyanin extraction. ^∗∗^*P* < 0.01 (Student’s *t*-test) and numbers indicate the ratio of anthocyanin levels (17°C → 28°C/17°C).

MYBL2 is a negative regulator of late anthocyanin biosynthetic genes such as *DFR* and *LDOX* (Dubos et al., 2008; Matsui et al., 2008). It has been shown that HY5 directly binds to the promoter of *MYBL2* and reduces its expression, thereby indirectly activating *DFR* and *LDOX* transcription (Wang et al., 2016). Since the level of the HY5 protein is reduced at high temperatures (**Figure 5D**), it is highly likely that *MYBL2* expression is also temperature-regulated. To examine this possibility, we compared *MYBL2* expression in seedlings grown at 17°C to that of seedlings grown at 17°C for 4 days followed by 28°C for 24 h. We found that *MYBL2* expression was increased about twofold by the high temperature treatment in wild type seedlings (**Figure 6A**). Interestingly, *MYBL2* expression was constantly high irrespective of ambient temperature in the *hy5* mutant, suggesting that the high temperature induced *MYBL2* expression through inactivation of HY5. This interpretation is consistent with a recent report showing that HY5 negatively regulates *MYBL2* expression, both directly and indirectly through miRNA858a activity (Wang et al., 2016). Given that the expression of *DFR* and *LDOX* is reduced at high temperatures, the expression patterns of *MYBL2* were consistent with its function as a transcriptional repressor of *DFR* and *LDOX* and further suggests that MYBL2 contributes to the temperature regulation of anthocyanin biosynthesis. Therefore, we determined the anthocyanin accumulation of *mybl2* loss-of-function mutant (*mybl2-1*) seedlings grown at two different ambient temperatures (17 and 17°C followed by 28°C). Anthocyanin accumulation was less affected by high temperature treatment (28°C) in *mybl2-1* mutant than in wild type seedlings and, as a result, the anthocyanin levels of the high temperature-treated *mybl2* seedlings were significantly higher than those of wild type treated with 28°C (**Figure 6B**). These results indicate that MYBL2 partially mediates the high temperature repression of anthocyanin biosynthesis. Therefore, it appears that high temperature represses anthocyanin biosynthesis through two distinct hierarchical, connected, and complementary mechanisms: (1) inactivation of the positive anthocyanin regulator HY5, and (2) transcriptional activation of *MYBL2*, which negatively regulates *DFR* and *LDOX* expression.

## Discussion

**FIGURE 7:**
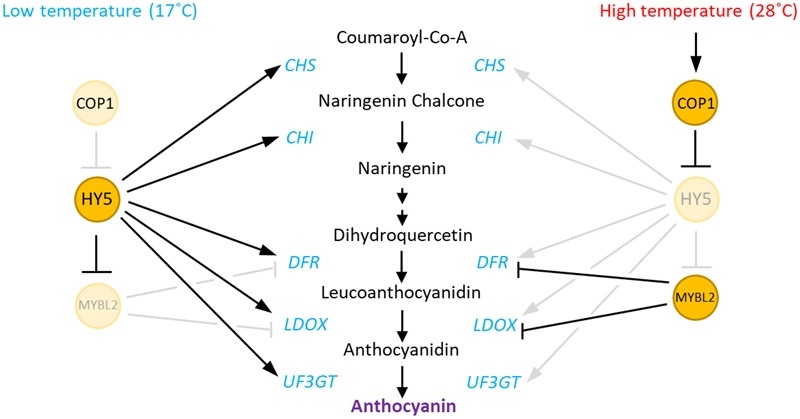
Hypothetical model of the regulation of anthocyanin biosynthesis by high temperature in *Arabidopsis.* At low ambient temperatures, HY5 directly activates the expression of both early (*CHS* and *CHI*) and late (*DFR, LDOX*, and *UF3GT*) anthocyanin biosynthetic genes. HY5 also indirectly represses the expression of the late genes by repression of *MYBL2*, which encodes a repressor of *DFR* and *LDOX*. In contrast, at high ambient temperatures, increased COP1 activity induces the degradation of HY5 protein, which results in low expression of both the early and late anthocyanin biosynthetic genes. The HY5 degradation also transcriptionally activates *MYBL2*, which also contributes the repression of the late genes at high temperatures.

In the present study, we demonstrated that anthocyanin biosynthesis is repressed by an elevation in ambient temperature and that a COP1-HY5 signaling module mediates the high-temperature regulation of anthocyanin biosynthesis. We propose that high temperature induces HY5 protein degradation through COP1, as recently suggested by Park et al. (2017), which directly leads to an induced expression of the negative regulator of anthocyanin biosynthesis (*MYBL2*), and reduced expression of both early (*CHS* and *CHI*) and late (*DFR, LDOX*, and *UF3GT*) anthocyanin biosynthetic genes. As a consequence, anthocyanin pigment levels are lower in seedlings grown at a high temperature (**Figure 7**).

High ambient temperature induces dramatic morphological changes in *Arabidopsis* seedlings, including the promotion of hypocotyl elongation and hyponastic leaf growth. PIF4 is a key regulator of the thermo-induced morphological changes and thus, hypocotyl elongation, in *pif4* mutant seedlings (and also in the *pif4;pif5* double mutant) is insensitive to high temperatures (Koini et al., 2009). In contrast to its thermo-insensitive hypocotyl elongation phenotype, anthocyanin accumulation and expression of anthocyanin biosynthetic genes in the *pif4;pif5* double mutant were significantly decreased in response to high temperatures. These results suggest that the anthocyanin biosynthesis is regulated by the high temperatures, independent of the PIF4-mediated morphogenic responses. Although the anthocyanin biosynthetic genes were down-regulated by high temperatures, basal levels of *CHS, DFR*, and *LDOX* were slightly higher in the *pif4;pif5* double mutant than in the wild type, indicating the negative functions of PIF4 and PIF5 in anthocyanin biosynthesis. In agreement with these observations, a previous study showed that these factors negatively regulate anthocyanin biosynthesis under red light (Liu et al., 2015).

Our results show that anthocyanin accumulation is thermo-insensitive in the *cop1-4* mutant. COP1 activity is known to be regulated by light receptor phytochromes (Yi and Deng, 2005). The light-activated phytochromes induce translocation of COP1 protein from the nucleus to the cytoplasm, thereby reducing the nuclear COP1 level; as a result, nuclear HY5 protein levels increase. Given that COP1 mediates the high temperature-induced degradation of HY5, it is likely that COP1 activity is increased at high temperatures. However, the expression of *COP1* was not significantly affected by high temperatures (Supplementary Figure 2), suggesting that COP1 is post-transcriptionally regulated by ambient temperature. In support of this hypothesis, a recent study reported that the nuclear COP1 protein level is increased at elevated temperatures, which is responsible for the high temperature-induced degradation of HY5 (Park et al., 2017). It was shown that the activities of phytochromes are regulated by ambient temperature as well as light (Jung et al., 2016; Legris et al., 2016). Phytochromes switch between an inactive Pr state and an active Pfr state depending on light conditions. Increased temperature promotes the reversion of active Pfr to the Pr state and the activities of phytochromes are decreased at high temperature (Jung et al., 2016; Legris et al., 2016). Therefore, it is likely that the high activity of COP1 at high temperature may be due to reduced phytochrome activities. Further experiments are required to determine if HY5 protein stability is regulated by temperature through the phytochrome-COP1 interacting module.

The accumulation of anthocyanin pigments in the ripening fruit of grapevine or apple has been reported to be reduced at high temperatures (Mori et al., 2007; Lin-Wang et al., 2011). In grapevine (*Vitis vinifera* L.), the expression of the HY5 homologous gene *VviHY5* is highly correlated with flavonol levels, and ectopic expression of *VviHY5* transcriptionally activates flavonol-related genes (*FLS4* and *GT5*) (Loyola et al., 2016), indicating a positive role for the HY5 ortholog in flavonol biosynthesis. In addition, a recent study proposed that VviHY5 may regulate anthocyanin biosynthesis by controlling *MYBA* genes (Matus et al., 2017). The expression of *VviHY5* increases in response to high temperatures under light conditions (Loyola et al., 2016). Even if *VviHY5* expression increases with high temperature, this may be irrelevant if the VviHY5 protein is also degraded as in *Arabidopsis*. Therefore, further studies are required to determine if the COP1-HY5 module mediates the high temperature repression of anthocyanin biosynthesis in other species.

Anthocyanin biosynthesis is increased at cold temperatures, and this increase has been suggested to enhance the survival of plants under cold stress (Catala et al., 2011). By contrast, the benefit of suppressing anthocyanin biosynthesis at high temperatures is unknown. Given that both morphological changes and anthocyanin biosynthesis are energy-demanding processes, the energy balance between these two processes might be optimized in response to the environmental conditions. In this respect, the suppression of anthocyanin biosynthesis might be more beneficial for plants grown at high temperature because thermomorphogenic growth at a cost of reduced anthocyanin biosynthesis might enhance survival under high temperature stress.

## Materials and Methods

### Plant Materials and Growth Conditions

*Arabidopsis thaliana* plants were grown in a greenhouse with 16 h light/8 h dark cycles at 20 to 24°C for general growth and seed harvesting. All the *A. thaliana* plants (*hy5, pif4;pif5, mybl2-1*, and *cop1-4*) used in this study were of the Col-0 ecotype background. The *hy5* mutants were obtained from the Salk Institute (Salk_ 056405C). The *pif4;pif5* seeds were previously described (Lorrain et al., 2008). The *cop1-4* mutant was previously described (McNellis et al., 1994). The *mybl2-1* mutant was previously described (Dubos et al., 2008). The *35S:HY5-GFP* (in Col-0 and *cop1-4* background) transgenic plants were described previously (Park et al., 2017).

### Gene Ontology (GO) Analysis

The list of high temperature-repressed genes for GO analysis was obtained from a previous RNA-seq experiment (Jung et al., 2016). In that experiment, genes with expression levels that were reduced more than four-fold at ZT0 in seedlings grown at 27°C compared to seedlings at 22°C were defined as high temperature-repressed genes. Low-expression genes [sum of FPKM values (22°C + 27°C) < 0.2] were excluded from the analysis. Enriched GO terms of the high temperature-repressed genes (a total of 915 genes) were identified using the Plant GeneSet Enrichment Analysis Toolkit (Yi et al., 2013) and heat maps for the expression of the anthocyanin biosynthetic genes were generated using Heatmapper web-based tools (Babicki et al., 2016).

### Anthocyanin Extraction

Seeds were sterilized using 70% (v/v) ethanol and 0.01% (v/v) Triton X-100 and were then plated on MS medium (Duchefa) containing 3% sucrose. After 3 days of stratification at 4°C, the plates were placed under white light for 6 h to promote seed germination. Seedlings were incubated at 17°C under continuous light conditions (light intensity: 30 μmol m^-2^ s^-1^) for 5 days or incubated at 17°C for 4 days followed by incubation at 28°C for 24 h. For anthocyanin extraction, 30 seedlings from each condition were placed in 600 μL of 1% HCl in methanol (v/v) (1% methanolic HCL = 45 mL methanol + 450 mL HCl). The samples were then incubated overnight in the dark at 4°C with shaking. After overnight incubation, 400 μL water and 400 μL chloroform were added to each sample and mixed. After centrifugation, the absorbance of the supernatant was measured at 530 and 657 nm, and the concentration of anthocyanin was calculated by *A*_530_ - 0.25^∗^*A*_657_ (Rabino and Mancinelli, 1986).

### qRT-PCR Gene Expressions Analysis

Seedlings were grown at 17°C for 5 days or grown at 17°C for 4 days followed by incubation at 28°C for 24 h. Seedlings (about 50 for each biological replicate) were then harvested and ground in liquid nitrogen for total RNA extraction. Total RNA was extracted from the seedlings using a MiniBEST Plant RNA extraction kit (TaKaRa). M-MLV reverse transcriptase (Fermentas) was used to synthesize cDNA from the RNA. Quantitative real-time PCR (qRT-PCR) was performed using a CFX96 Real-Time PCR detection system (Bio-Rad) and the EvaGreen master mix (Solgent). Gene expression levels were normalized to that of *PP2A* (*PROTEIN PHOSPHATASE 2A SUBUNIT*, AT1G13320) and are shown relative to the expression levels in wild type (Czechowski et al., 2005). Three biological replicates were analyzed for each qRT-PCR experiment. The gene specific primers used here are listed in Supplementary Table 2.

### Protein Extraction and Western Blot Analysis

Five-day-old seedlings (about 50 seedlings in total) were harvested and ground in liquid nitrogen. Proteins were extracted with 2× protein extraction buffer (100 mM Tris-HCl, pH 6.8, 25% glycerol, 2% SDS, 0.01% bromophenol blue, with β-mercaptoethanol added to 10% before use). Western blot analysis was performed to determine HY5-GFP protein levels using an anti-GFP antibody (Santa Cruz, sc-9996). The original images for the western blot and Ponceau S staining are shown in Supplementary Figure 3.

## Author Contributions

SK, EO, and GH conceived and designed the research, performed the experiments, and wrote the manuscript, SL, J-YZ, TN, and IP performed the experiments and analyzed the data, JK wrote the manuscript.

## Conflict of Interest Statement

The authors declare that the research was conducted in the absence of any commercial or financial relationships that could be construed as a potential conflict of interest. The reviewer NA and handling Editor declared their shared affiliation.
