# Supplementary figures and images for: High Ambient Temperature Represses Anthocyanin Biosynthesis through Degradation of HY5
Source: Front Plant Sci. 2017 Oct 20;8:1787. doi: 10.3389/fpls.2017.01787 (PMC5655971; doi:10.3389/fpls.2017.01787)

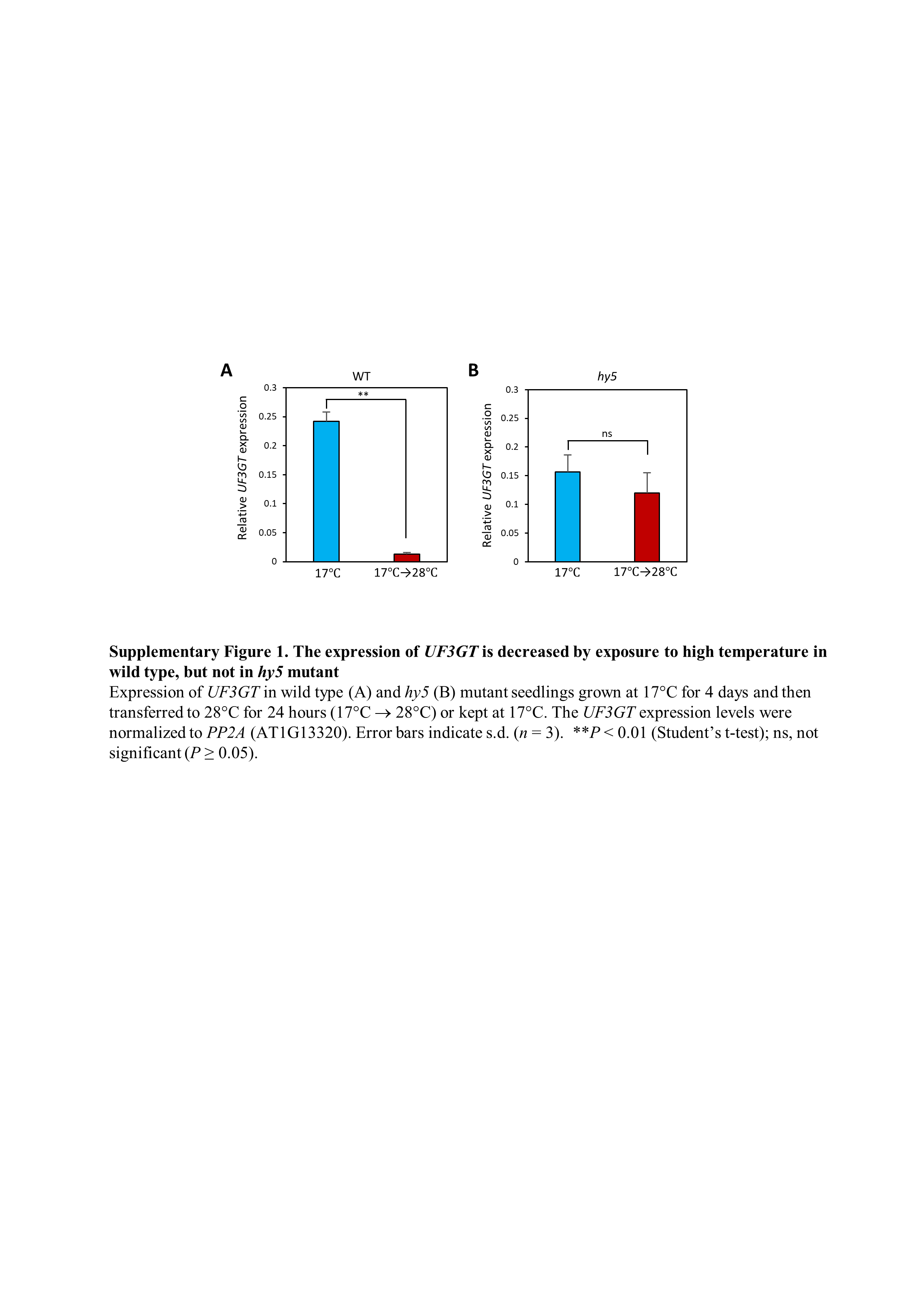

Supplement: Supplementary file 3 [file Image_1.TIF]

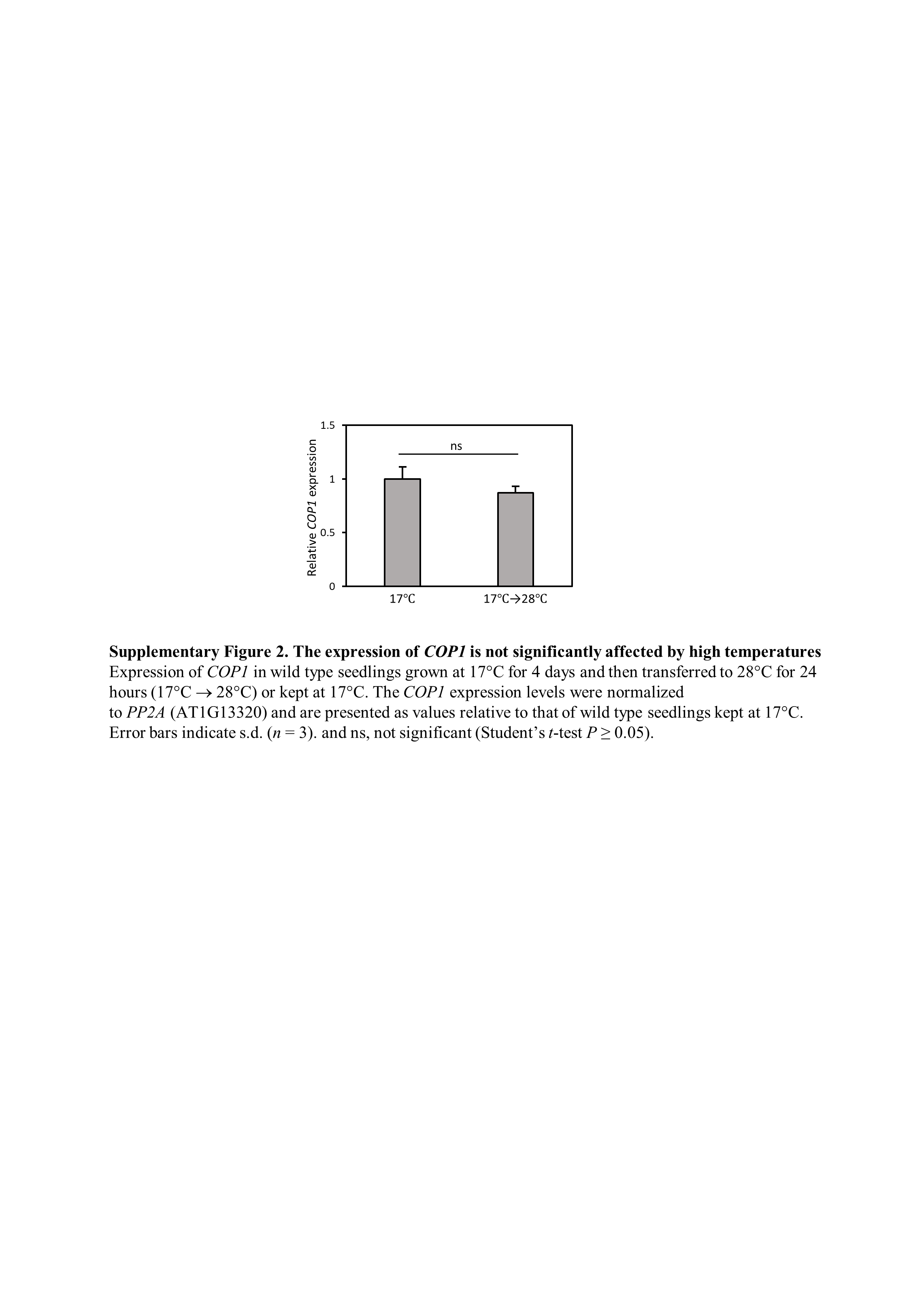

Supplement: Supplementary file 4 [file Image_2.TIF]

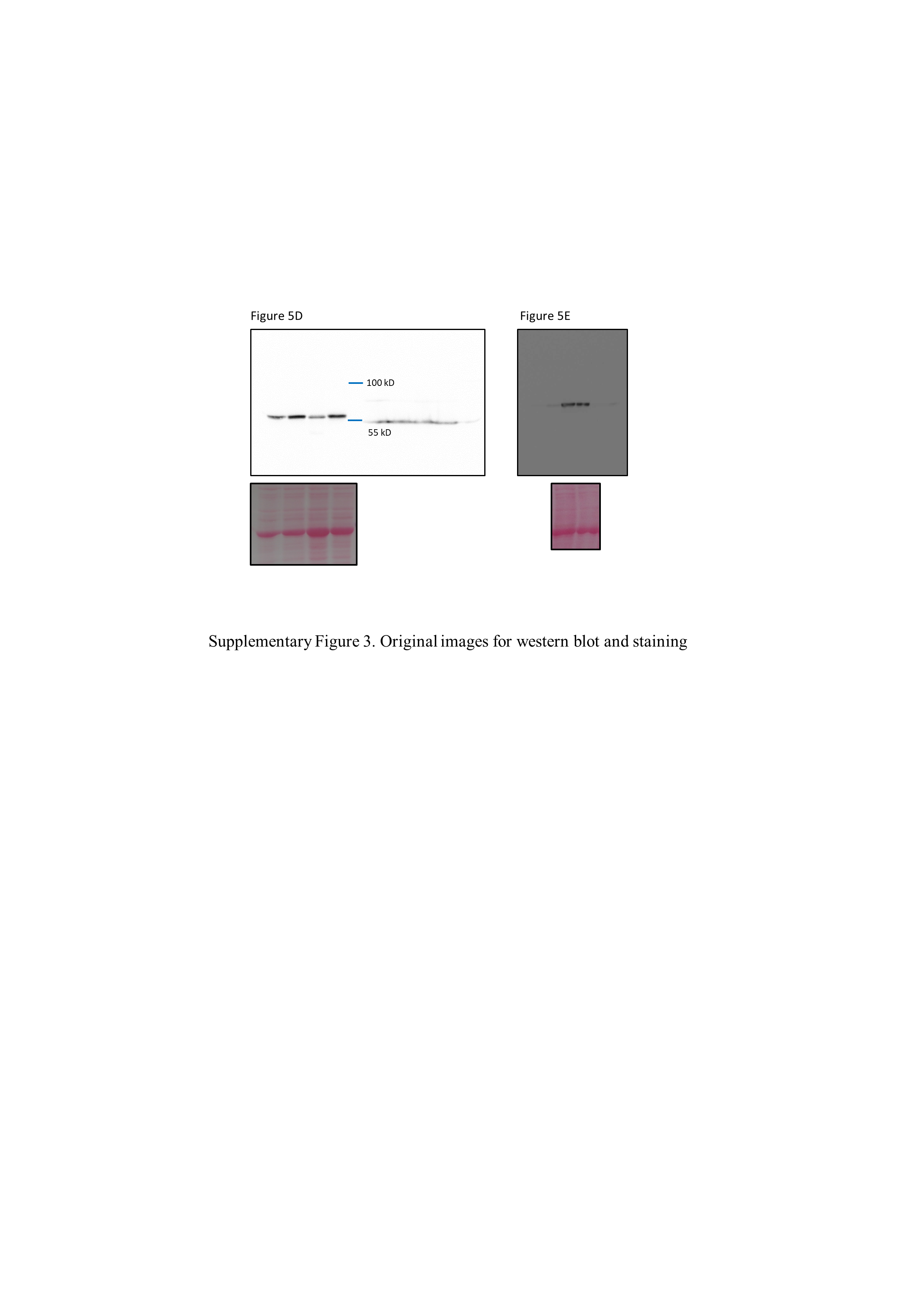

Supplement: Supplementary file 5 [file Image_3.TIF]
